# Narrow Bottlenecks Affect *Pea Seedborne Mosaic Virus* Populations during Vertical Seed Transmission but not during Leaf Colonization

**DOI:** 10.1371/journal.ppat.1003833

**Published:** 2014-01-09

**Authors:** Frédéric Fabre, Benoît Moury, Elisabeth Ida Johansen, Vincent Simon, Mireille Jacquemond, Rachid Senoussi

**Affiliations:** 1 INRA, UR407 Pathologie Végétale, Domaine Saint Maurice, Montfavet, France; 2 University of Copenhagen, Department of Plant and Environmental Sciences, Frederiksberg C, Denmark; 3 INRA, UR546 Biostatistique et Processus Spatiaux, Domaine Saint-Paul, Site Agroparc, Avignon, France; University of Toledo, United States of America

## Abstract

The effective size of populations (*Ne*) determines whether selection or genetic drift is the predominant force shaping their genetic structure and evolution. Populations having high *Ne* adapt faster, as selection acts more intensely, than populations having low *Ne*, where random effects of genetic drift dominate. Estimating *Ne* for various steps of plant virus life cycle has been the focus of several studies in the last decade, but no estimates are available for the vertical transmission of plant viruses, although virus seed transmission is economically significant in at least 18% of plant viruses in at least one plant species. Here we study the co-dynamics of two variants of *Pea seedborne mosaic virus* (PSbMV) colonizing leaves of pea plants (*Pisum sativum* L.) during the whole flowering period, and their subsequent transmission to plant progeny through seeds. Whereas classical estimators of *Ne* could be used for leaf infection at the systemic level, as virus variants were equally competitive, dedicated stochastic models were needed to estimate *Ne* during vertical transmission. Very little genetic drift was observed during the infection of apical leaves, with *Ne* values ranging from 59 to 216. In contrast, a very drastic genetic drift was observed during vertical transmission, with an average number of infectious virus particles contributing to the infection of a seedling from an infected mother plant close to one. A simple model of vertical transmission, assuming a cumulative action of virus infectious particles and a virus density threshold required for vertical transmission to occur fitted the experimental data very satisfactorily. This study reveals that vertically-transmitted viruses endure bottlenecks as narrow as those imposed by horizontal transmission. These bottlenecks are likely to slow down virus adaptation and could decrease virus fitness and virulence.

## Author Summary

Short generation times and high mutation rates are the hallmarks of virus. They favor their fast adaptation as illustrated by their ability to overcome natural as well as man-made barriers such as host resistance or drug treatments. However, such a fast adaptation could be slowed down when genetic drift, which introduces random sampling effects in the evolution of virus populations, is important. Whether genetic drift or selection dominates depends on the effective size of populations (*Ne*). *Ne* has been estimated for several steps of plant virus infectious cycle, such as horizontal transmission by insects and the colonization of plant cells and tissues. However, although economically important, no estimate of *Ne* during vertical transmission of viruses, *i.e.* the infection of plant progenies from parental plants, is available. Here, we report that *Pea seedborne mosaic virus* (PSbMV), a seed transmitted virus infecting pea crops, undergoes very drastic genetic drift during vertical transmission, with an average number of infectious virus particles contributing to the infection of a seedling from an infected mother plant close to one. Such bottlenecks, as narrow as those imposed by horizontal transmission, could slow down virus adaptation and should be taken into account to improve plant protection strategies.

## Introduction

Evolution of virus populations depends on several forces including mutation, recombination, genetic drift, selection and migration, acting concomitantly but exerting pressures that vary widely in direction and intensity. It makes therefore difficult to predict viral emergences or the durability of control strategies. The relative intensity of these forces will determine whether evolution follows predominantly stochastic or deterministic patterns. The concept of effective size of populations, *Ne*, plays a core role since it determines the rate of random fluctuations of the frequency of virus variants caused by genetic drift across generations in a model population. *Ne* estimates the number of individuals that pass on their genes through generations. It is usually much smaller than the total size of populations: although the total size of virus populations in their host plants can be tremendous and reach 10^7^ to 10^9^ virus particles [1], [2], estimates of *Ne* are below 500 and most of them are actually close to one [3], [4]. Importantly, for populations affected by periodic size changes like bottlenecks or founder effects, *Ne* is given by the harmonic mean of population sizes over generations [5]. As a consequence, even short periods of small population size during the life cycle or history of populations can have disproportionately strong influences on *Ne*. *Ne* helps to predict the loss and distribution of neutral genetic variation [6], the fixation probabilities of beneficial or deleterious alleles [7], and the fitness and survival of small populations [8]. Therefore, knowledge of *Ne* is of major interest for modeling disease emergence and can be an important issue in agriculture as illustrated by the breakdown of plant resistance genes by adapted virus variants [9], [10].

It has been shown recently that plant virus populations undergo transient and recurrent bottlenecks at different steps of their life cycle, like during horizontal transmission, *i.e.* plant inoculation by vectors [9], [11], by contact with an infected plant [12] or by artificial inoculation [13], or during the colonization of plant cells [14], [15], [16] and tissues [4], [13], [15], [17]. By contrast, no estimates of bottleneck sizes during vertical transmission of plant viruses, *i.e.* infection of plant progenies by the parental plant(s), are available yet.

There are three major ways of vertical transmission of plant viruses *via* the contamination of true seeds. In only a few examples, particularly stable viruses such as tobamoviruses can be retained in the seed coat and then transmitted to the seedling after germination [18]. In that case, there is no contamination of the embryo and the process of seedling infection resembles horizontal transmission through contact with an infected plant. The two other ways of contamination correspond to invasion of the embryo by the virus, either from infected maternal tissues or, more rarely, via infected pollen. Although seed embryos are usually protected against invasion by viruses that affect the mother plant, many viruses have the capacity to circumvent this barrier. Even low rates of seed transmission can be epidemiologically important because secondary spread of viruses can begin as soon as the germination stage [19] and virus seed transmission can be economically significant for at least 18% of plant viruses [20].

The goal of this work was to compare the size of bottlenecks affecting populations of *Pea seedborne mosaic virus* (PSbMV) (genus *Potyvirus*, family *Potyviridae*) in pea plants during vertical seed transmission and during the colonization of leaves.

## Materials and Methods

### Plant and virus material

The PSbMV isolate DPD1 and the variant DPD1-R only differ at codon position 116 in the VPg (Virus protein genome-linked)-coding region were used. Codon 116 is GTG (valine) and CGA (arginine) in DPD1 and DPD1-R, respectively [21], and these three adjacent nucleotide differences allowed identification and quantification of the two PSbMV variants in mixed-infected plants (see below).

The pea (*Pisum sativum* L.) cultivar ‘Vedette’ that transmits PSbMV through seeds at high frequencies [22] was used for all experiments. No pollen transmission of PSbMV was observed in this genotype [23]. Plants were grown under greenhouse conditions from November 2011 to April 2012.

### Quantification of PSbMV variants in inocula and pea leaves

**Figure 1:**
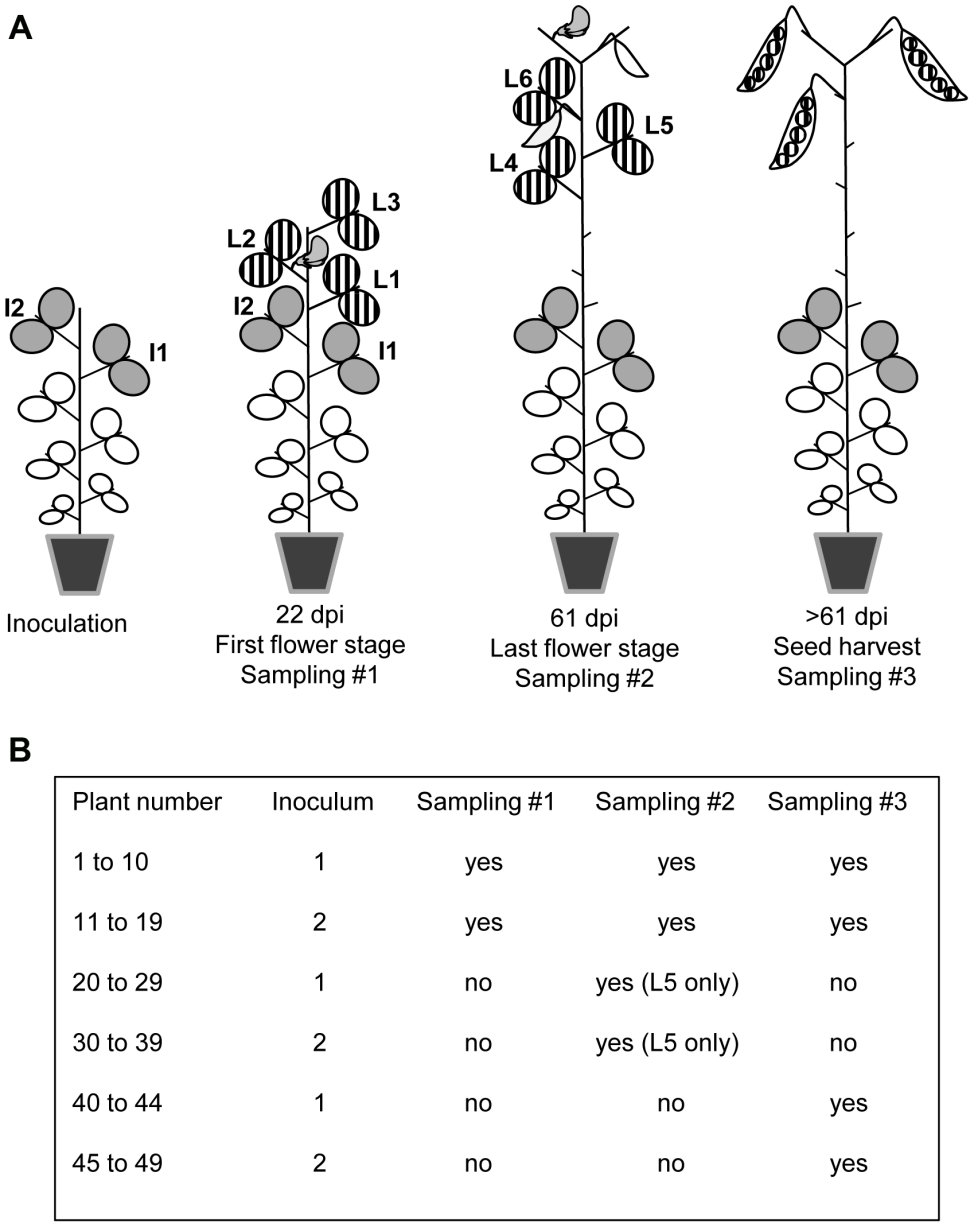
Virus sampling design for pea plants inoculated with PSbMV. (A) Plants of the pea cultivar Vedette were mechanically inoculated with mixtures of two PSbMV variants 28 days after sowing on the two leaves I1 and I2. Twenty-two days post inoculation (dpi), corresponding to the anthesis of the first flower in the plant population, the three leaves L1 to L3 immediately above I2 were collected separately and analyzed. Sixty-one dpi, corresponding to the end of anthesis, the three leaves L4 to L6 immediately above L3 were collected separately and analyzed. Finally, all pods produced by the main stem of the plants were harvested at desiccation step, seeds were sown and seedlings were analyzed 22 days after sowing. (B) Different sets of plants were subjected to different sampling schemes. For plants numbered 20 to 49, samplings at 22 dpi and/or at 61 dpi were omitted.

DPD1 and DPD1-R isolates were multiplied separately in Vedette plants and mixed at two different ratios, corresponding to 1∶1 and 1∶4 weights of infected leaf material, to create inocula 1 and 2, respectively. For each inoculum, 25 Vedette plants were inoculated 28 days after sowing (7 to 8 expanded leaf stage) on the two upper expanded leaves (Fig. 1A). All plants were mechanically inoculated. The Vedette plants were then split into three sets corresponding to three different leaf and seed sampling designs, and randomized. For inoculum 2, one plant died before leaf sampling. For plants numbered 1 to 19, leaves were collected at two different dates (Fig. 1B). At 22 days post inoculation (dpi), corresponding to the anthesis of the first flower in the plant population, the three leaves immediately above the inoculated ones were collected separately (leaves L1 to L3, Fig. 1A) and at 61 dpi (end of flowering), the three leaves immediately above leaf L3 were collected separately (leaves L4 to L6, Fig. 1A). For plants 20 to 39, only leaf L5 was collected (at 61 dpi). Finally, no leaves at all were collected on plants 40 to 49.

For inoculation, RNA extraction and enzyme-linked immunosorbent assay (ELISA), leaf tissue was homogenized in four volumes (wt/vol) of 0.03 M phosphate buffer (pH 7.0) supplemented with 2% (wt/vol) diethyldithiocarbamate. For RT-PCR, total RNA was extracted from a 150 µL aliquot using the Tri Reagent kit (Molecular Research Center Inc., Cincinnati, OH, USA). To amplify the VPg coding region that contained the polymorphic codon between DPD1 and DPD1-R, reverse transcription (RT) was performed on 2 µL of each RNA extract using *Avian myeloblastosis virus* reverse transcriptase (Promega Corp., Madison, WI, USA) followed by polymerase chain reaction (PCR) with *Thermus aquaticus* DNA polymerase (Promega Corp.). Primer DPD1-VPGR (5′-AAACTGACCAAATCCGATGCC complementary to nucleotides 6690 to 6710 of DPD1 genome, accession number D10930) was used for RT and primers DPD1-VPGR and DPD1-VPGF (5′-AAAACACTGCAGCTTAAGGG corresponding to nucleotides 5868 to 5887) were used for PCR. The PCR program started with 3 min at 95°C followed by 35 cycles (45 s at 95°C, 30 s at 55°C, 50 s at 72°C) and a final extension at 72°C for 10 min. Amplification products were sequenced directly with primer DPD1-VPGF by Genoscreen (Lille, France). We estimated the relative proportions of the two PSbMV variants in inocula and leaves from the height of peaks corresponding to the three polymorphic codon positions in the chromatograms. The reliability of this quantification method was evaluated with artificial mixtures of known quantities of the two PSbMV variants obtained after virus purification. As illustrated in Fig. S1, a linear regression allowed a very accurate prediction of the percentage of each variant in mixed-infected leaves (slope = 1.01, R^2^ = 0.99). This chromatogram-based quantification method was also compared to another method based on the cloning of RT-PCR products obtained with primers DPD1-VPGR and DPD1-VPGF into an *Escherichia coli* plasmid vector. For this, 5 pea leaves with contrasted frequencies of variant DPD1-R (from 24% to 69% based on the “chromatogram” method) were chosen and, for each of them, the RT-PCR products were cloned into the pGEM-T Easy vector (Promega Corp., Madison, WI, USA) and the number of clones corresponding to DPD1 and DPD1-R among a total of 40 clones per leaf was determined using the specific primers DPD1-VPgF-116V and DPD1-VPgF-116R described below. Again, the “chromatogram” and “cloning” methods provided highly similar frequency estimates (slope = 1.07, R^2^ = 0.96), hence further validating the “chromatogram” quantification method.

### Determination of seed transmission rates of PSbMV variants

Pods produced by the main stem (Fig. 1) of plants 1 to 19 and 40 to 49 were harvested at desiccation time. Harvested seeds were then sown and all leaves from each seedling were collected 22 days later. Seedling extracts were tested for PSbMV infection by antigen coated plate-ELISA (ACP-ELISA) using an antiserum specific for the PSbMV coat protein. To detect the presence of either the DPD1 or the DPD1-R PSbMV variants, total RNA was extracted from seedlings of mother plants with a minimum of nine ELISA-positive seedlings. The generic DPD1-VPGR primer was used for RT and for PCR in combination with either the primer DPD1-VPgF-116V (5′-CTCGATAAACAATTGTTTGTG ) or the primer DPD1-VPgF-116R (5′-CTCGATAAACAATTGTTTCGA ) corresponding to nucleotides 6336–6356 of DPD1 andDPD1-R, respectively. The PCR programs started with 3 min at 95°C followed by 40 cycles (45 s at 95°C, 30 s at 63°C, 30 s at 72°C) and a final extension at 72°C for 10 min. Artificial mixtures of known proportions of RNAs of the two PSbMV variants obtained after virus purification [9] were used to evaluate the sensitivity of the RT-PCR method. In these artificial mixtures, each variant could be detected up to a 0.1% relative concentration.

### Estimation of effective population size during leaf colonization

To estimate *Ne* during PSbMV colonization of upper uninoculated leaves (L1 to L6 in Fig. 1A), we used the “variance method” based on the differences in the variance of the viral genotype frequencies between the two sampling dates at 22 and 61 dpi, and the “*F*_ST_ method” based on the difference between these 2 dates of Wright's *F*_ST_ statistics [24] calculated on within- and between-plant viral genetic diversities [25]. These methods are based on the assumption that the PSbMV variants within the viral population under consideration are equally competitive.

According to the variance method, *Ne* = E(P)×(1−E(P))/[Var(P′)−Var(P)], where P and P′ are the random variables of the frequencies of the viral marker for each plant at the first and second sampling dates, respectively, E(P) is the expected value of P in the plant population and Var(P) its variance. In practice, E(P) and Var(P) were estimated by the sample mean and variance of the frequencies of the viral marker measured on a set of plants (Table 1). Because Var(P) was negligible compared to E(P) in our datasets, the *Ne* estimates provided by this equation were almost identical to those obtained with equation (14) of [26]: *Ne* = [E(P)×(1−E(P))−Var(P)]/[Var(P′)−Var(P)]. According to the *F*_ST_ method, *Ne* = (1−*F*_ST_)/(*F*_ST_′−*F*_ST_), where *F*_ST_ and *F*_ST_′ are values of the *F*_ST_ statistics of the viral populations at the first and second sampling dates, respectively (see [25] for details). For both methods, *Ne* confidence intervals were obtained by bootstrapping 10,000 times among plants.

**Table 1:** *Ne* estimates for the systemic colonization of pea leaves by PSbMV between 22 and 61 days post inoculation (dpi).

Dataset	*p_i_*: estimate of the frequencies of the viral marker at date 1 (22 dpi) in plant *i*	*p_i_′*: estimate of the frequencies of the viral marker at date 2 (61 dpi) in plant *i*	*Ne* estimation (“variance method”)		*Ne* estimation (“*F*_ST_ method”)	
			Inoculum 1	Inoculum 2	Inoculum 1	Inoculum 2
1	Average of 3 leaves *j* per plant *i* a 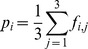	Average of 3 leaves *j* per plant *i* a 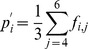	NA^c^ [150–10,954]	92 [33–1,238]	NA^c^ [171–12,970]	85 [39–1,772]
2	Average of 3 leaves *j* per plant *i* a 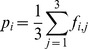	One leaf chosen randomly per plant *i* a 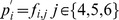	197 [77–4,270]	59 [32–323]	216 [83–5,153]	67 [37–335]
3	Average of 3 leaves *j* per plant *i* a 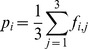	Leaf L5 for plants *i* b 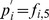	133 [59–1,463]	74 [28–993]	143 [64–1,511]	82 [31–1,129]

Estimates were obtained by two different methods and separately for two inocula corresponding to two different initial ratios of PSbMV variants. 95% confidence intervals estimated by bootstrapping among plants are indicated in brackets. The variable *f_i,j_* is the relative frequency of virus variant 1 in plant *i* and leaf *j* (*j* in {1,2,3} for date 1 and *j* in {4,5,6} for date 2) (see Fig. S1 for details on its estimation).

^a^For inocula 1 and 2, *i*∈[1–10] and *i*∈[11–19], respectively.

^b^For inocula 1 and 2, *i*∈[20–29] and *i*∈[30–39], respectively.

^c^The variance and *F*_ST_ methods assume an increase of the variance of viral frequencies (respectively of the *F*ST statistics of viral populations) with time. “NA” (not available), indicates situations where these assumptions were not satisfied and, consequently, where genetic drift was negligible (*Ne* tends to infinity).

With the nested sampling design used (several leaves being analyzed for each plant) and with the different plants sets available (plants 1 to 19, analyzed at 22 and 61 dpi and plants 20 to 39 analyzed at 61 dpi only, Fig. 1B), several datasets can be used to estimate *Ne* (Table 1). All leaves can be considered to estimate the variant frequencies at date 2 and *Ne* reflects the overall genetic drift process in the whole plant (dataset 1) or a single leaf per plant can be considered at date 2 (as in [25]) and, in that case, *Ne* can be viewed as the number of founding virus particles contributing to the colonization of an individual leaf (datasets 2 and 3). In addition, different sets of plants can be considered for each date (dataset 3) to test the influence of sampling leaves at date 1 on *Ne* estimates (by comparing dataset 2 and dataset 3).

### Estimation of the size of population bottlenecks during PSbMV seed transmission

In order to estimate the size of bottlenecks undergone by PSbMV populations during seed transmission and to explore the mechanisms underlying seed transmission, we developed dedicated models. These models describe the two sequential processes leading to seedling infection: (1) virus entry into the seed (or more precisely into seed embryos, see the Discussion section) and (2) seedling infection from the contaminated seed. Concerning the first step, we assumed that the two virus variants act independently and, for a given variant, virus particles also act independently (*i.e.* there is no variant-variant nor virus-virus interactions). Concerning the second step, both types of interactions were considered (Table 2).

**Table 2:** Models for virus vertical transmission.

Seedling infection status	No seedling infection	Seedling infection by variant 1 only	Seedling infection by variant 2 only	Seedling infection by both variants	Is seedling infection density dependent?	Is there variant-variant interactions?
**M1**: Additive action for vertical transmission	*N^1^*+*N^2^*≤*N^c^*	*N^1^*>*N^c^* and *N^2^* = 0	*N^1^* = 0 and *N^2^*>*N^c^*	*N^1^*+*N^2^*>*N^c^*, *N^1^*>0 and *N^2^*>0	Yes	Yes ; interchangeable^a^
**M2**: Additive action for vertical transmission with low level of inhibition between variants	*N^1^*+*N^2^*≤*N^c^*	*N^1^*+*N^2^*>*N^c^*, *N^2^*≤*N^c^* and *N^1^*>*N^2^*	*N^1^*+*N^2^*>*N^c^*, *N^1^*≤*N^c^* and *N^2^*>*N^1^*	(*N^1^*>*N^c^* and *N^2^*>*N^c^*) or (*N^1^*+*N^2^*>*N^c^* and *N^1^* = *N^2^*)	Yes	Yes ; not interchangeable^a^
**M3**: Independent action for vertical transmission	*N^1^*≤*N^c^* and *N^2^*≤*N^c^*	*N^1^*>*N^c^* and *N^2^*≤*N^c^*	*N^2^*>*N^c^* and *N^1^*≤*N^c^*	*N^1^*>*N^c^* and *N^2^*>*N^c^*	Yes	No
**M4**: No threshold for seedling infection	*N^1^* = 0 and *N^2^* = 0	*N^1^*>0 and *N^2^* = 0	*N^2^*>0 and *N^1^* = 0	*N^1^*>0 and *N^2^*>0	No	No

*N^1^* (respectively *N^2^*) is the number of PSbMV variant 1 (DPD1-R) (respectively variant 2 (DPD1)) particles entering a given seed of a given plant and *N^c^* is a critical threshold for the infection of the seedling issued from this seed. Note that for *N^c^* = 0 the models M1, M2 and M3 are identical to model M4.

^a^When variant-variant interactions occur, two cases were distinguished depending on whether, or not, virus variants are interchangeable whatever their type. Variants are interchangeable for seedling infection if the contribution to seedling infection of a virus particle of one variant does not depend on the density of virus particles of the other variant.

For the first step (virus entry into the seed), we assumed that the proportions of PSbMV variants DPD1-R (variant 1) and DPD1 (variant 2) in coinfected plants can fluctuate in time during the period of seed infection (*i.e.* from 22 to 61 dpi) and within the plant because of spatial heterogeneity of distribution of virus variants. We considered that the relative frequencies *f^1^* of variant 1 and *f^2^* = (*1−f^1^*) of variant 2 in the vicinity of a given seed at infection time were realization of random variables that followed Beta distributions of parameters (α,β) and (β,α), respectively, α and β varying from plant to plant. We assumed that the numbers of viral particles of each variant entering a given seed, *N^1^* and *N^2^*, were described by independent Poisson processes of parameters λ^1^×*f^1^* and λ^2^×(*1−f^1^*), respectively, where λ^1^ and λ^2^ are the efficiencies of seed infection by variants 1 and 2. This hypothesis implies that all virus particles of a given variant have the same probability of entering a seed, and that they enter into the seeds independently of each other (*i.e*. there is no virus-virus interactions). Moreover, assuming that these Poisson processes are independent implies that there is no interaction between DPD1 and DPD1-R variants for entering a seed (however they can enter with different efficiencies).

**Figure 2:**
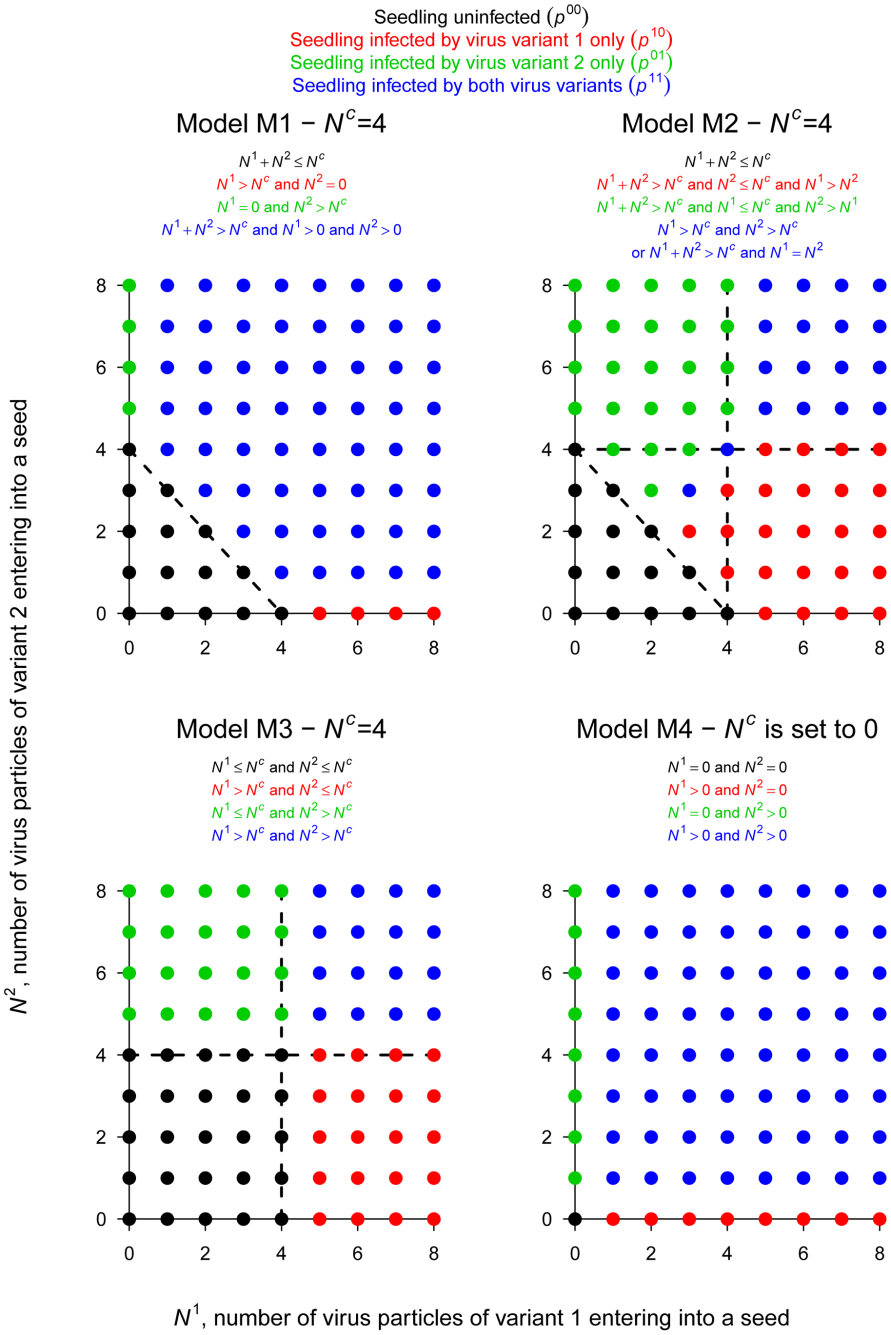
The 4 models of PSbMV vertical transmission. This figure illustrates the 4 sets of infection rules governing vertical transmission (*i.e.* seedling infection) and corresponding to the 4 models considered here (models M1, M2, M3 and M4). For each model, the rules leading to the 4 possible categories of seedling infection ((i) healthy, (ii) infected only by variant 2 (DPD1), (iii) infected only by variant 1 (DPD1-R) and (iv) infected by both PSbMV variants) are indicated and illustrated for values of *N^1^* and *N^2^* ranging from 0 to 8 and *N^c^* = 4. Let remember that where *N^1^* (resp. *N^2^*) is the number of particles of type 1 (resp. 2) entering into the seed and *N^c^* is a threshold for efficient seedling infection.

For the second step of PSbMV seed transmission (seedling infection), we hypothesized that vertical transmission occurs if a minimal number *N^c^*+1 of viral particles entered into a seed. *N^c^* was chosen randomly and independently for each seed (and plant) from a Poisson distribution of parameter λ^c^. Four alternative models were considered to describe the mechanism of seedling infection (Table 2, Fig. 2). Models M1, M2 and M3 assume virus-virus interactions, seedling infection being a virus density-dependent process. In models M1 and M2, variant-variant interactions occur, as seedlings become infected if the total number of particles of virus variants 1 and 2 entering into a seed (*i.e. N^1^*+*N^2^*) strictly exceeds *N^c^*. In model M1, a variant is transmitted vertically if at least one particle of this variant has entered into the seed, meaning that the contribution to seedling infection of a virus particle of one variant does not depend on the density of the other variant: virus particles are interchangeable, whatever their type. In contrast, in model M2, a variant is transmitted vertically if its density is higher than *N^c^* or higher than the density of the other variant (when *N^1^* = *N^2^* the seedling becomes infected by both variants). Here, the contribution of a virus particle of one variant to seedling infection depends on the density of the other variant: virus particles are not interchangeable and model M2 assumed some inhibition between variants when one variant outnumbers the other. In model M3, there is no variant-variant interaction: the virus variants initiate seedling infection independently. A variant is transmitted vertically if the number of particles of this variant entering into the seed strictly exceeds *N^c^*. Finally, in model M4 there is no virus-virus, nor variant-variant interaction. *N^c^* is indeed set to zero: a virus variant is transmitted vertically if at least one particle of this virus variant has entered the seed.

The R plants of the experimental design, indexed by *r*, were assumed to be independent. For a given plant, the variables describing the infection status of the seedlings, indexed by *s* (1≤*s*≤*S_r_*), were supposed to be independent and identically distributed, but potentially with different distributions, for distinct plants. The variable 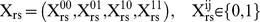 with 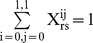, describes the infection status of seedling *s* issued from mother plant *r*. This seedling is either not infected (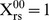), infected only by variant 1 (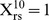), infected only by variant 2 (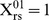), or infected by both variants (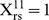). 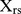 defines a categorical (or 1-trial multinomial) variable. Let 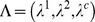 for model M1, M2 and M3 or 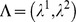 for model M4 and 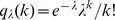 be the probability density function (pdf) of Poisson distribution, and let 
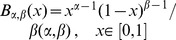
 be the beta pdf of the random variable 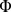 standing for the proportion of variant 1 circulating into the phloem.

For seedling *s* of plant *r*, if the proportion 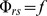
of variant 1 present in the circulating viral population is known, the conditional probabilities of the different seedling infection statuses are denoted 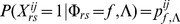.

For model M1, we have:
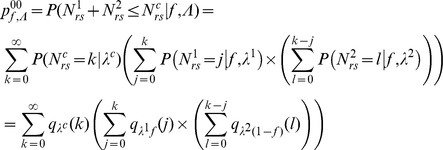


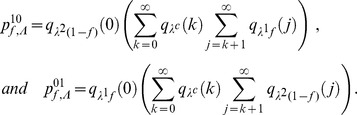


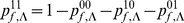
For model M2, we have:
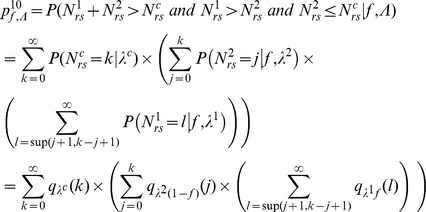


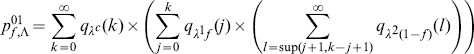
The formula for 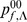 given for model M1 is the same for model M2.

For model M3, we have:
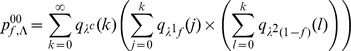


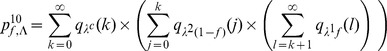


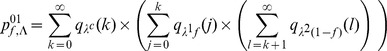
Finally, for model M4, we have:
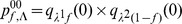


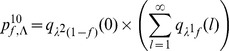


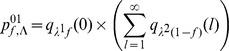
Since only the variables 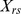 are observed in the experiments but neither 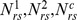 nor 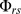, the likelihood of observing 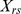 is obtained by integrating over the values of variable Φ. In our case, the plant specific parameters (*α_r_*, *β_r_*) were considered as known parameters and have been estimated for a given plant using the proportions of the two virus variants in leaves L1, L2 and L3 at 22 dpi and in leaves L4, L5 and L6 at 61 dpi (Fig. 1). Since the realized frequencies *f* were not observed, the probability 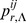 for a seedling of a given plant *r* to be in the infectious status *ij* is obtained by integrating over all possible realizations of 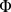, that is 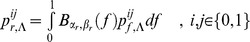.

The likelihood of a given model 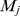 is obtained as the product of R multinomial distributions as
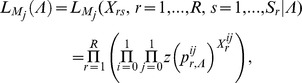
where 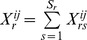 with 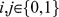 since the X_rs_ are independent for the different plants.

After checking that the four models were practically identifiable in our experimental conditions (Text S1), model parameter inferences were performed by minimizing the log of the likelihood function 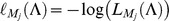 for each model M_j_ using the “bbmle” package with the “nlminb” optimization routines of the R software environment (http://cran.r-project.org/). 95% confidence intervals for model parameters were estimated using the function “profile” of the “bbmle” package.

## Results

To analyze genetic drift and bottlenecks affecting virus populations during leaf infection and seed transmission, we inoculated plants of *Pisum sativum* cv. Vedette with two mixtures of the PSbMV variants DPD1 and DPD1-R (inocula 1 contained 38% of variant DPD1-R and inocula 2 contained 66% of variant DPD1-R) and we examined the composition of the viral populations at two time points in apical leaves of the inoculated plants, and in the plant progeny issued from the seeds collected on these mother plants. Changes in frequency of the PSbMV variants during the infection process and in the seedling progeny could be due either to genetic drift, selection or both. Since many models used to estimate *Ne* assume that changes of genotype frequencies in populations are due to genetic drift only, and not to selection, we tested whether the marker that allows distinguishing DPD1 from DPD1-R was neutral, *i.e.* if the two variants were equally competitive.

### The two PSbMV variants are equally competitive for systemic movement in leaves

To estimate the relative competitiveness of PSbMV variants DPD1 and DPD1-R for infection of leaves at the systemic level, we compared their relative frequencies in apical leaves sampled at 22 and 61 dpi (Fig. 1). Analysis *a posteriori* based on the sequence chromatograms (Fig. S1) indicated that the DPD1-R variant represented 37.8% and 65.9% of inocula 1 and 2, respectively (the method used to estimate the relative frequency of the two PSbMV variants in inocula and mixed-infected leaves is described in details in Fig. S1). Sequence chromatograms of the VPg coding region showed also clearly that both PSbMV variants were present in each of the 134 leaves examined at 22 and 61 dpi (for plants 1 to 19) or at 61 dpi only (for plants 20 to 39) (Fig. 1B). Indeed, at the sequence region polymorphic between DPD1 and DPD1-R, the lowest of the six peaks (two different nucleotides for each of the three polymorphic codon positions) was 3.0 to 9.5 times (5.0 times on average) higher than the highest peak of background noise. The minimum and maximum percentages of DPD1-R among the 134 leaves were 21.3 and 70.6%, respectively. At 22 dpi, the mean proportion of DPD1-R observed in three sampled leaves was 32.3% for inoculum 1 and 55.7% for inoculum 2 (Table 3). In the same plants at 61 dpi, these average proportions were 31.0% and 51.7%, respectively, indicating almost no change in average frequency between the two dates and equal competitiveness of the two viral variants during leaf colonization. Confirming this, the difference of variant proportions in the plants between the two dates was 2.5% on average (with a 5.4% standard deviation). It was lower than 5% for 16 of the 19 analyzed plants. Twelve plants showed a decrease and seven an increase of DPD1-R frequency, which is not significantly different from random fluctuations (*P* = 0.25; Wilcoxon matched pairs signed ranks test). In addition, the sampling at 22 dpi had no influence on the average composition of the viral populations. Indeed, the average proportions of DPD1-R frequency in plants sampled only at 61 dpi (plants 20 to 39) were 31.4% and 58.2% for inocula 1 and 2, respectively, which is not significantly different from the DPD1-R frequencies at 22 or 61 dpi in plants that were sampled twice (plants 1 to 19) (*P*>0.20; Mann-Whitney tests) (Table 3).

**Table 3:** Frequency of two PSbMV variants in pea leaves and seedlings in three sets of plants corresponding to three sampling designs.

Plants	1 to 19						20 to 39	40 to 49			
	Leaves^a^		Seedlings^b^				Leaves^a^	Seedlings^b^			
	22 dpi	61 dpi	(i)	(ii)	(iii)	(iv)	61 dpi	(i)	(ii)	(iii)	(iv)
Inoculum 1	*n* = 30	*n* = 30	*n* = 378				*n* = 10	*n* = 180			
	32.3 (4.1)	31.0 (3.7)	63.8	15.6	10.3	10.3	31.4 (5.8)	64.1	19.8	9.5	6.6
Inoculum 2	*n* = 27	*n* = 27	*n* = 450				*n* = 10	*n* = 188			
	55.7 (5.7)	51.7 (7.6)	66.9	6.7	16.2	10.2	58.2 (8.0)	65.5	7.5	15.4	11.7

For plants 1 to 19, leaves were sampled at 22 and 61 dpi; for plants 20 to 39, leaves were sampled only at 61 days post inoculation (dpi) and for plants 40 to 49, no leaves were sampled. *n*: total number of leaves or seedlings analyzed. Seedlings were analyzed only for plants that produced nine seeds or more. The percentages of the DPD1-R variant in inocula 1 and 2 were 37.8% and 65.9%, respectively.

^a^Mean relative frequency (×100) and standard deviation (×100; between parentheses) of the DPD1-R specific marker in the viral population.

^b^Frequency of healthy seedlings (i), of seedlings infected by DPD1 (ii) or DPD1-R (iii) only, and of seedlings co-infected by both PSbMV variants (iv).

Consequently, the two PSbMV variants DPD1 and DPD1-R can be considered as equally competitive with regard to the colonization of leaves at the systemic level between 22 and 61 dpi.

### Evidence for slightly higher seed transmissibility for DPD1-R over DPD1

To estimate the relative competitiveness of PSbMV variants DPD1 and DPD1-R for seed transmission, we compared their relative frequencies in seedlings derived from inoculated mother plants and in leaves of these mother plants sampled at 22 and 61 dpi.

The number of harvested pea seeds in the different PSbMV-infected plants varied from seven to 95, with an average of 54. All harvested seeds germinated and the infection status of each seedling was analyzed by ELISA. From a total of 1022 seedlings derived from mother plants 1 to 19, the average seedling infection rate was 33.4% (33.1% and 33.7% for inocula 1 and 2 respectively). The average seedling infection rate was also similar to the infection rates observed in the seedlings of control plants inoculated by DPD1-R only (36% for a total of *n* = 206 seedlings) or DPD1 only (31%; *n* = 195), (*P* = 0.27; Khi^2^ tests). Accordingly, the fact that similar percentages of seed transmission were observed in single-infected or mixed-infected plants suggests independence between PSbMV variants for seed infection and justifies the Poissonian assumptions made for modeling virus entry into seeds. Finally, the seedling infection rates were similar to those observed for mixed-infected plants for which no leaves were sampled (plants 40 to 49) (34%; *n* = 584; *P*>0.30 for both inocula; Khi^2^ tests). All these values are in the range of seed transmission rates obtained independently with PSbMV DPD1 and Vedette pea plants (25 to 53% seed transmission [27]).

For mother plants having nine or more infected seedlings, the proportion of seedlings corresponding to categories (ii) seedling infected only by variant DPD1, (iii) seedling infected only by variant DPD1-R and (iv) seedling infected by both PSbMV variants was determined (Table 3). Accordingly, among plants 1 to 19, the seedlings obtained from 12 plants (six plants initially inoculated with 38% of variant DPD1-R (inoculum 1) and six plants initially inoculated with 66% of variant DPD1-R (inoculum 2)) were analyzed. In contrast to plant leaves, the two PSbMV variants were detected simultaneously in a minority of infected seedlings, *i.e*. 28.5% of seedlings for inoculum 1 and 30.9% of seedlings for inoculum 2. The DPD1-R variant was observed in 39.8% and 70.9% of seedlings infected by a single virus variant (considering only seedling categories (ii) and (iii)) for inocula 1 and 2, respectively. Compared to the PSbMV variant frequencies in the leaves of the mother plants, DPD1-R seemed to be somewhat better seed-transmitted than DPD1, a difference which is significant only for inoculum 2 (*P* = 0.01; Khi^2^ test). Examining seed transmission results for each mother plant individually did not reveal any significant difference between the distributions of variants among the seedlings and the average proportion of PSbMV variants in leaves.

The percentages of seedlings infected simultaneously by the two PSbMV variants were similar for mother plants which leaves were sampled twice (numbers 1 to 19) and for mother plants for which no leaves were sampled (plants 40 to 49) (*P*>0.2; Khi^2^ tests) (Table 3). The distributions of the two PSbMV variants among the seedlings were also similar for these two sets of mother plants (*P*>0.2; Khi^2^ tests) (Table 3). Consequently, the sampling procedure did not affect the seed transmission of the PSbMV variants and will not bias the estimates of bottleneck sizes during PSbMV seed transmission.

### Small genetic drift effects during the systemic colonization of pea leaves by PSbMV

Since the two inoculated PSbMV variants DPD1 and DPD1-R were equally competitive during the colonization of plant leaves from 22 to 61 dpi, we used the methods described in [25] to estimate *Ne*. These methods are based on the differences in variance of the viral variant frequencies (“variance method”) or on the difference of Wright's *F*_ST_ statistics (“*F*_ST_ method”) between two sampling dates. For these methods, an underlying assumption is that the variance of the viral variant frequencies (or the *F*_ST_ statistics) increases with time. Indeed, considering that all variants are equally fit in the population, variant frequency fluctuations are due only to genetic drift, which affects both the amount and distribution of neutral genetic diversity over time (*i.e*. across generations) and space (*i.e.* between subpopulations at a given time).

Whatever the datasets used, we observed very small differences of variance of virus variant frequencies or *F*_ST_ statistics for the PSbMV populations at 22 and 61 dpi, suggesting very limited effect, if any, of genetic drift on viral populations during the systemic invasion of apical leaves (Table 1). Accordingly, *Ne* estimates ranged from 59 to 216, with a mean of all *Ne* estimates of 111 and 119 for the variance and *F*_ST_ methods, respectively, and of 172 and 77 for inocula 1 and 2, respectively. In some cases, no *Ne* estimates could be obtained because the variance of viral frequencies and *F*_ST_ statistics decreased between 22 and 61 dpi (no drift was observed). Overall, little difference was observed between the “variance” and “*F*_ST_” methods and between the different datasets used to estimate viral frequencies at the two dates of observations (Table 1). Notably, leaf sampling at date 1 did not affect significantly the results: *Ne* estimates were comprised between 74 and 143 for dataset 3 (independent sets of plants were sampled at each date) and between 59 and 216 for dataset 2 (the same set of plants was sampled at both dates) (Table 1). Dataset 3 provided the most homogeneous *Ne* estimates and smallest confidence intervals.

Bootstrapping among plants allowed obtaining confidence intervals for *Ne* estimates. The 95% confidence intervals were large because of the small number of plants and because the small differences in virus frequency variances or population *F*_ST_ between dates 1 and 2 had large impacts on *Ne* estimates (Table 1). All these results demonstrated the lack of narrow population bottlenecks during the leaf colonization at the systemic level, and provided *Ne* estimates similar to those obtained for CaMV [25].

### Narrow bottlenecks affect PSbMV populations during vertical seed transmission

The “variance” and “*F*_ST_” methods provide unbiased estimates of *N_e_* only if the variants analyzed are equally competitive. This is not the case for our vertical transmission dataset, as variant DPD1-R was somewhat better transmitted to seedlings than DPD1. Thus, we developed stochastic models to estimate the size of bottlenecks undergone by PSbMV populations during seed transmission that (i) take into account the difference in seed transmissibility between variants and (ii) that allow to disentangle different seedling infection processes (see the Materials, methods and models section). These models showed that the mean number of PSbMV particles contributing to the infection of an individual seedling was close to one. We first checked whether our experimental design (number and nature of the data) was sufficiently informative to estimate accurately the model parameters using practical identifiability tests (Text S1). Numerical simulations indicated clearly that all four models had a very good practical identifiability. Indeed, whatever the parameters considered, the coefficient of correlation between their true and estimated values were ≥0.94 (Table 4). Moreover, the four models of virus seed transmission could be very efficiently discriminated using Akaike Information Criterion (AIC) [28]. When the data were simulated under model 1, the AIC selected model 1 (respectively models 2, 3 and 4) in 92% (respectively 2%, 6% and 0%) of simulations. Similarly, when the data were generated under models 2, 3 or 4, the AIC identified the correct model in 94%, 100% and 88% of the simulations.

**Table 4:** Practical identifiability of virus seed transmission models.

	Parameters		
Model	λ^1^	λ^2^	λ^c^
M1	0.98 (0.002)	0.99 (0.002)	0.98 (0.004)
M2	0.97 (0.005)	0.97 (0.005)	0.95 (0.01)
M3	0.95 (0.01)	0.94 (0.012)	0.95 (0.01)
M4	0.99 (0.001)	0.99 (0.001)	not applicable

Correlation coefficients (and in brackets their standard deviations estimated with a bootstrapping method) between the true and estimated parameter values for the 4 models (Table 2) of virus seed transmission (over 100 simulated datasets).

The model selection procedure applied to the experimental data set (Table S1) indicates that the AIC values of models M1 to M4 were 195, 196, 203 and 207, respectively. The corresponding Akaike weights, which provide the relative support for each model, were 0.59, 0.40, 0.01 and nearly zero (10^−17^). Thus, although model M1 is supported best by the data, model M2 has also a substantial support [29]. Assuming that λ^1^ = λ^2^, the AIC of the models increased to 207, 210, 219 and 279 for M1, M2, M3 and M4, respectively, indicating that the mean number of viruses contributing to the infection of a seedling was significantly different for virus variants 1 and 2. Under model M1, parameter inference indicated that the mean number of DPD1-R variant infectious particles contributing to the infection of a pea seedling was 1.08 (with a 95% confidence interval, CI_95%_, ranging from 0.9 to 1.29) and 0.74 for virus variant DPD1 (with a CI_95%_ ranging from 0.61 to 0.88), while the mean number of virus particles required to infect a pea seedling was 0.84 (CI_95%_ = [0.63, 1.05]). Parameter inferences under model M2 (which is almost as likely as model M1 with an Akaike weight of 0.4) were close to those obtained with M1, although always slightly higher: 1.52 for λ^1^ with a CI_95%_ ranging from 1.08 to 2.74, 1.06 for λ^2^ with a CI_95%_ ranging from 0.75 to 1.95 and 1.36 for λ^c^ with a CI_95%_ ranging from 0.85 to 2.63.

Importantly, models M1 and M2 fitted very satisfactorily the experimental data. First, the observed and predicted mean numbers of seedlings corresponding to the four categories of seedling infection (*i.e.* (i) healthy, (ii) infected only by variant 2 (DPD1), (iii) infected only by variant 1 (DPD1-R) and (iv) infected by both PSbMV variants) were highly correlated (R^2^ = 0.88) for both models. Second, between-plant variability was very well represented, as an 80% (resp. 90%) confidence interval predicted by model M1 contained 78% (resp. 83%) of the observed data. For model M2, an 80% (resp. 90%) predicted confidence interval contained 80% (resp. 89%) of the observed data.

## Discussion

We used the PSbMV-pea pathosystem to estimate the size of bottlenecks affecting a plant virus population during vertical transmission through seed embryo. We observed a very drastic genetic drift during vertical transmission, with an average number of infectious virus particles contributing to the infection of a seedling from an infected mother plant close to one. On the opposite, almost no genetic drift was observed during the infection of apical leaves of the mother plants during the same time-frame corresponding to the flowering period.

Estimation of *Ne* during the infection cycle of plant virus populations is quite complicated because of (i) the lack of estimates of generation times for viruses [30], which is due to the difficulties inherent to the definition of a viral generation (different lengths of time may be required for the production of the different components of progeny virus particles, like structural proteins and genome components), to the overlap between replication of virus entities within populations, and to the complex kinetics of virus replication [31] and (ii) the succession of different steps in the virus infection cycle that potentially follow different growth dynamics (intracellular accumulation, cell-to-cell movement, systemic translocation and plant-to-plant transmission). In spite of these limitations, several estimates of *Ne* or of the bottleneck size corresponding to particular steps of the virus life cycle have been obtained.

Concerning the colonization of plant leaves by viruses, estimates obtained for *Ne* are quite contrasted [3], [4]. The low genetic drift (large *Ne*) observed during the systemic colonization of pea plants by PSbMV corroborates previous results obtained with *Cauliflower mosaic virus* (genus *Caulimovirus*) [3], [25] or *Tobacco etch virus* (genus *Potyvirus*) [4], where the composition of virus populations were compared between inoculated and apical leaves [4] or between apical leaves sampled at two different dates [25]. In contrast, small *Ne* values were obtained by comparing the virus populations between the inoculum and apical leaves [13], [17]. These observations were reconciled by showing that most genetic drift occurs at the inoculation step whereas little genetic drift is subsequently observed during the systemic colonization of plants [4]. However, genetic drift during the systemic colonization of plants by viruses is not necessarily low. For example, 13 years after inoculation, each leaf of a peach tree was colonized by a single viral variant of *Plum pox virus* (PPV, genus *Potyvirus*) whereas a total of 33 viral variants were observed in the whole set of leaves analyzed, indicating that narrow bottlenecks acted on PPV populations during the infection of individual leaves [32]. Clearly, additional studies are needed to unravel the plant, virus and environmental factors which determine the patterns and intensity of genetic drift during plant colonization by viruses. Recently, the number of virus colonizing leaves was shown to increase with the concentration of viruses circulating within the plant sap [33]. This suggests that the low level of genetic drift observed during the systemic colonization of pea plants by PSbMV during the flowering period could result from high concentrations of virus circulating into the plant vasculature.

On the opposite, during the same time-frame, we showed that a single infectious PSbMV particle contributed on average to the infection of an individual seedling derived from an inoculated mother plant. To our knowledge, this is the first estimate of the bottleneck size imposed by vertical transmission to a virus population. Strong bottlenecks were also observed during vertical mother-to-child transmission of *Human immunodeficiency virus*-1 (HIV-1) [34], [35], [36]. For the majority of *in utero* or intrapartum transmission cases examined in these three studies (65%; 22/49) the infants harbored a single viral variant, which suggested the occurrence of narrow population bottlenecks at transmission. Note, that this percentage is very close to our own estimates for PSbMV (we observed from 66% to 82% single-infected pea seedlings among the infected ones, depending on inocula and plant sets; Table 3). A recent study conducted on seed transmission of ZYMV (*Zucchini yellow mosaic virus*, genus *Potyvirus*) in *Cucurbita pepo* showed that 16 of 24 ZYMV variants present in the mother plant were also present in vertically-transmitted virus populations, either of the first or second plant generation [37]. These figures suggest that bottleneck sizes during vertical transmission could be larger in that case. However, in none of these studies was the transmissibility of the different virus variants or their abundance in the mother's plasma (or in the mother plant) taken into account, which hampers the derivation of unbiased estimates for the bottleneck size.

Vertical transmission of PSbMV occurs through the infection of the pea seed embryos [38]. Usually, viruses are excluded from plant reproductive tissues. Because pathogens must cross several barriers intended to protect the developing embryo, the occurrence of narrow population bottlenecks during pathogen vertical transmission could be a quite general rule. The capacity of viruses to invade plant embryos and withstand seed maturation and desiccation depends both on virus and host genotypes, as demonstrated for PSbMV [23], [27], [39]. Seed transmission of PSbMV in pea occurs exclusively by direct invasion of immature embryos from virus-infected maternal tissues. It occurs only during a precise temporal window and from virus accumulated at a precise location in the developing seed. Such conditions are therefore favorable to the occurrence of strong virus population bottlenecks. Early infection of the mother plant is necessary for PSbMV vertical transmission to occur [23]. PSbMV invasion of pea embryos occurs from virus infection spreading from the maternal cells in the micropylar region of the embryo to the endosperm cytoplasm, then to the embryonic suspensor and finally to the embryo. Since the embryonic suspensor undergoes a programmed cell death, it acts for the virus as a “transient conduit” for embryo invasion [39]. The ability of the virus to invade the micropylar region before the suspensor programmed cell death therefore explains why early PSbMV infection of the mother plant is required for seed transmission, and could also explain why some pea cultivars are resistant to PSbMV seed transmission and why some PSbMV isolates are not seed transmitted in pea. In addition, no PSbMV replication could be detected in the endosperm cytoplasm [38], suggesting that only a small amount of virus is able to accumulate into the endosperm cytoplasm and further enter the suspensor. Based on these observations, Roberts et al [38] suggested that seed transmission of PSbMV was largely based on the chance of the virus to be in the right place at the right time. In these conditions, even a small degree of heterogeneity in the distribution of virus variants in the cells of infected plants, as observed for some potyviruses [16], [31], [40], could contribute to the genetic drift that occurs during PSbMV seed transmission. In agreement with these observations, the models that we used to estimate the bottleneck size during seed transmission considered that the virus variant frequency could fluctuate randomly at the time and place of virus entry into seed embryos. Consequently, the biological processes involved in PSbMV seed transmission are in accordance with, and provide plausible mechanisms for the narrow bottlenecks endured by virus populations during vertical seed transmission. To go further, it would be worth investigating whether the virus load in plants is linked to the intensity of genetic drift during the colonization of leaves, as evidenced by [33], and whether it affects also genetic drift during vertical transmission, at least at some critical time points during embryo infection.

From a methodological point of view, the mathematical framework introduced here allowed disentangling the relative importance of selection and genetic drift in shaping the genetic composition of viral populations during seed transmission. It could be of broad interest to estimate *Ne* when the effect of deterministic evolutionary forces, typically selection, cannot be excluded. Indeed, the temporal methods classically used to estimate *Ne* assume that the observed changes in allele frequency are due to genetic drift only and thus require the use of neutral genetic markers for the population of interest [6]. Such markers could be difficult to identify or to generate, especially for viruses, which typically possess highly constrained genomes and are impacted by strongly negative average mutational effects on fitness [41]. From a biological point of view, model selection analysis indicated that seedling infection was a virus density-dependent process, where particles of the two virus variants sum up their action to exceed an infection threshold, rather than a process where each variant acts independently (models M1 and M2 were preferred to model M3). These results echo the study of Lafforgue *et al.* [42], who showed that the delay of systemic infection of a plant was determined by the cumulative effect of independently-acting foci of primary infection. Results also showed that one or a small number of viral particle(s) is (are) enough for virus seed transmission (as *λ^c^* was low), indicating that each virus particle has a quite high probability of causing efficient seed transmission. However, rejection of model M4 indicates that one viral particle is not always sufficient to initiate efficient seedling infection. Model M1 being only slightly preferable to model M2, it remained unclear whether virus particles belonging to the two variants are interchangeable or not in the cumulative infection process, interchangeability meaning that the contribution to seedling infection of a virus particle of one variant does not depend on the density of virus particles of the other variant. Consequently, more data should be gathered to clearly distinguish whether frequency-dependent selection of PSbMV variants occurred (as in model M2) or not (as in model M1) during seed transmission.

The small *Ne* values observed for PSbMV vertical transmission are expected to impact more deeply virus evolution than bottlenecks of the same size that would be experienced during horizontal transmission [9], [11], at least for large host populations. This is suggested by theoretical work on the evolution of parasites virulence (defined as the harm that they inflict to their host) according to their mode of transmission. The classical mechanism to explain why vertically-transmitted parasites evolve reduced virulence is through an indirect selection to improve host survival and/or reproduction [43]. Our study suggests that such reduced virulence could also be the consequence of narrow bottlenecks during vertical transmission. Indeed, using a model that assumed a tight association between parasites fitness and virulence, Bergstrom et al. [44] suggested that a direct effect of narrow bottlenecks is to select much lower levels of virulence in vertically-transmitted than in horizontally-transmitted pathogens. This was mainly due to the decrease of intra-host competition between virus variants in case of vertical transmission. Said another way, the strength of selection is reduced in case of vertical transmission as virus particles are separated into many distinct evolutionary host lineages. In their study, this difference between vertical and horizontal transmission was particularly strong when only one or two virus particles initiate the infection of a new host. In agreement with these theoretical results, repeated vertical transmission events were shown to affect drastically the evolution of PSbMV populations. As soon as the second generation of pea plants contaminated by PSbMV through seed transmission, PSbMV populations derived from four different isolates were shown to differ largely from the initial inocula: in contrast to the initially inoculated plants (generation 0), or plants of the first generation issued from contaminated seeds, the infected plants of the second generation did not express any symptom and PSbMV was not detectable in their vegetative parts [45]. Such a rapid evolution could be, at least in part, a consequence of the severe bottlenecks experienced by PSbMV populations during vertical transmission. Similar declines in virulence [46] or symptom induction [37], [47], [48] have been observed for other seed-transmitted plant viruses.

Exploring to which extent such decrease in virulence or symptomatology (two life history traits that are not necessarily correlated in plant viruses) can be explained by bottleneck sizes is an important issue in parasite evolution. From an applied perspective, many vertically-transmitted plant viruses are also transmitted horizontally by vectors. For example, PSbMV is transmitted by a large number of aphid species. In the field, ecological (*e.g*. host density, aphid population dynamics) and agronomic factors (*e.g* use of virus-free seeds) determine which mode of transmission is prevailing. Undoubtedly, a deeper understanding of the balance between the relative importance of these transmission modes during the course of epidemics, coupled with a deeper knowledge of the bottleneck sizes associated with these transmission modes is needed to better understand the evolution of important pathogen life history traits such as virulence, symptom severity and yield losses. Ultimately, this research could help designing more efficient strategies of plant protection relying on the knowledge and manipulation of evolutionary changes in parasites populations.

## Supporting Information

Figure S1 **Quantification of the frequency of two PSbMV variants in mixed-infected pea leaves.** The two PSbMV variants DPD1 and DPD1-R were purified separately from infected Vedette plants according to the protocol described by [9], quantified spectrophotometrically, and mixed in known ratios (artificial mixtures containing 10, 20, 40, 50 or 80% of DPD1-R) with an extract of leaves of healthy pea plants (0.5 g of leaves ground in four volumes (wt/vol) of 0.03 M phosphate buffer (pH 7.0) supplemented with 0.2% (wt/vol) diethyldithiocarbamate) at a final concentration of 10 ng/µl of virus (x-axis). From these PSbMV solutions, RNA extractions and RT-PCR were performed in triplicate as described in the Materials and Methods section. PCR products were sequenced directly and the relative proportion of the PSbMV DPD1-R variant (y-axis) was estimated from the height of the peaks in the sequence chromatograms with the following formula: 1/3×[H(_346_C)/[H(_346_C)+H(_346_G)]+H(_347_G)/[H(_347_G)+H(_347_T)]+H(_348_A)/[H(_348_A)+H(_348_G)]], where H(_z_X) is the height of the peak corresponding to nucleotide X at position z of the PSbMV VPg cistron on the sequence chromatogram. DPD1 and DPD1-R possess a GTG (respectively CGA) codon at position 116 (*i.e*. nucleotide positions 346, 347 and 348) of the VPg cistron. In the graph, the relative proportion of the PSbMV DPD1-R variant is plotted as a function of the known proportion of the PSbMV DPD1-R variant in the artificial mixture for the 3 replicates realized.
(PDF)

Table S1
**Data set used to estimate the size of bottlenecks during PSbMV seed transmission.** The seedlings obtained from 12 plants (six for inoculum 1 with 38% of variant DPD1-R and six for inoculum 2 with 66% of variant DPD1-R - *i.e.* only from mothers plants having nine of more infected seedlings) were analyzed by ELISA and PSbMV-variant-specific RT-PCR in order to distinguishing four categories of seedlings: (i) healthy, (ii) infected by DPD1, (iii) infected by DPD1-R and (iv) infected by both PSbMV variants. In all, 828 seedlings have been analysed. For each plant, the mean and standard deviation of the relative frequency of DPD1-R variants during the flowering period were estimated using in all 6 leaves, the 3 leaves sampled at 22 dpi and the 3 sampled at 61 dpi. For each plant *r* (1≤*r*≤12), these mean and standard deviation estimated were used to calculate the parameters 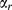 and 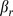 of the Beta distribution modeling the variability of the proportion of virus variant DPD1-R during the time of seed infection (see Text S1).(PDF)

Text S1 **Identifiability of the virus vertical transmission models.**
(PDF)
