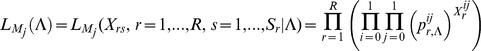# Correction: Narrow Bottlenecks Affect *Pea Seedborne Mosaic Virus* Populations during Vertical Seed Transmission but not during Leaf Colonization

**DOI:** 10.1371/journal.ppat.1004468

**Published:** 2014-09-23

**Authors:** The PLOS Pathogens Staff

In the Materials and Methods section, there is an error in the equation in the section titled “Estimation of the size of population bottlenecks during PSbMV seed transmission” that appears immediately after the phrase “The likelihood of a given model *M_j_* is obtained as the product of R multinomial distributions as.”

The “z” should not appear in the equation. Please view the complete, correct equation here: