# Supplementary material for: Narrow Bottlenecks Affect Pea Seedborne Mosaic Virus Populations during Vertical Seed Transmission but not during Leaf Colonization
Source: PLoS Pathog. 2014 Jan 9;10(1):e1003833. doi: 10.1371/journal.ppat.1003833 (PMC3887104; doi:10.1371/journal.ppat.1003833)

Figure S1. Quantification of the frequency of two PSbMV variants in mixed-infected pea leaves. The two PSbMV variants DPD1 and DPD1-R were purified separately from infected Vedette plants according to the protocol described by [9], quantified spectrophotometrically, and mixed in known ratios (artificial mixtures containing 10, 20, 40, 50 or 80% of DPD1-R) with an extract of leaves of healthy pea plants (0.5 g of leaves ground in four volumes (wt/vol) of 0.03 M phosphate buffer (pH 7.0) supplemented with 0.2% (wt/vol) diethyldithiocarbamate) at a final concentration of 10 ng/µl of virus (x-axis). From these PSbMV solutions, RNA extractions and RT-PCR were performed in triplicate as described in the Materials, methods and models section. PCR products were sequenced directly and the relative proportion of the PSbMV DPD1-R variant (y-axis) was estimated from the height of the peaks in the sequence chromatograms with the following formula:

$1/3\times[H(_{346}C)/[H(_{346}C)+H(_{346}G)]+H(_{347}G)/[H(_{347}G)+H(_{347}T)]+H(_{348}A)/[H(_{348}A)+H(_{348}G)]]$, where $H(_zX)$ is the height of the peak corresponding to nucleotide X at position z of the PSbMV VPg cistron on the sequence chromatogram. DPD1 and DPD1-R possess a GTG (respectively CGA) codon at position 116 (*i.e.* nucleotide positions 346, 347 and 348) of the VPg cistron. In the graph, the relative proportion of the PSbMV DPD1-R variant is plotted as a function of the known proportion of the PSbMV DPD1-R variant in the artificial mixture for the 3 replicates realized.

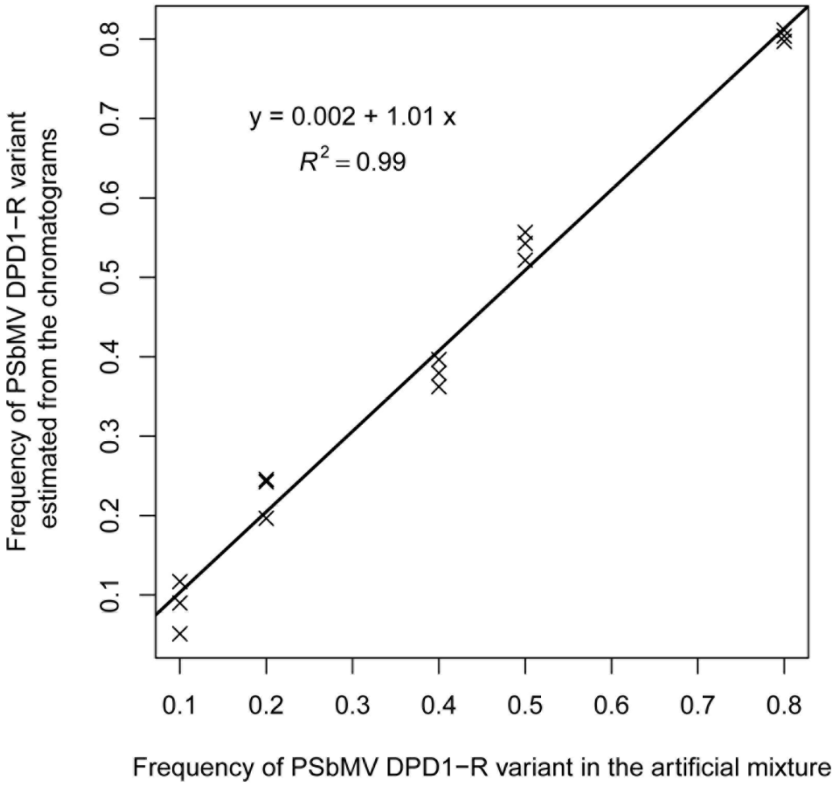

Supplement: Figure S1 — Quantification of the frequency of two PSbMV variants in mixed-infected pea leaves. The two PSbMV variants DPD1 and DPD1-R were purified separately from infected Vedette plants according to the protocol described by [9], quantified spectrophotometrically, and mixed in known ratios (artificial mixtures containing 10, 20, 40, 50 or 80% of DPD1-R) with an extract of leaves of healthy pea plants (0.5 g of leaves ground in four volumes (wt/vol) of 0.03 M phosphate buffer (pH 7.0) supplemented with 0.2% (wt/vol) diethyldithiocarbamate) at a final concentration of 10 ng/µl of virus (x-axis). From these PSbMV solutions, RNA extractions and RT-PCR were performed in triplicate as described in the Materials and Methods section. PCR products were sequenced directly and the relative proportion of the PSbMV DPD1-R variant (y-axis) was estimated from the height of the peaks in the sequence chromatograms with the following formula: 1/3×[H(346C)/[H(346C)+H(346G)]+H(347G)/[H(347G)+H(347T)]+H(348A)/[H(348A)+H(348G)]], where H(zX) is the height of the peak corresponding to nucleotide X at position z of the PSbMV VPg cistron on the sequence chromatogram. DPD1 and DPD1-R possess a GTG (respectively CGA) codon at position 116 (i.e. nucleotide positions 346, 347 and 348) of the VPg cistron. In the graph, the relative proportion of the PSbMV DPD1-R variant is plotted as a function of the known proportion of the PSbMV DPD1-R variant in the artificial mixture for the 3 replicates realized. (PDF) [file ppat.1003833.s001.pdf]
