# Supplementary material for: Narrow Bottlenecks Affect Pea Seedborne Mosaic Virus Populations during Vertical Seed Transmission but not during Leaf Colonization
Source: PLoS Pathog. 2014 Jan 9;10(1):e1003833. doi: 10.1371/journal.ppat.1003833 (PMC3887104; doi:10.1371/journal.ppat.1003833)
Supplement: Table S1 — Data set used to estimate the size of bottlenecks during PSbMV seed transmission. The seedlings obtained from 12 plants (six for inoculum 1 with 38% of variant DPD1-R and six for inoculum 2 with 66% of variant DPD1-R - i.e. only from mothers plants having nine of more infected seedlings) were analyzed by ELISA and PSbMV-variant-specific RT-PCR in order to distinguishing four categories of seedlings: (i) healthy, (ii) infected by DPD1, (iii) infected by DPD1-R and (iv) infected by both PSbMV variants. In all, 828 seedlings have been analysed. For each plant, the mean and standard deviation of the relative frequency of DPD1-R variants during the flowering period were estimated using in all 6 leaves, the 3 leaves sampled at 22 dpi and the 3 sampled at 61 dpi. For each plant r (1≤r≤12), these mean and standard deviation estimated were used to calculate the parameters and of the Beta distribution modeling the variability of the proportion of virus variant DPD1-R during the time of seed infection (see Text S1). (PDF) [file ppat.1003833.s002.pdf]

Table S1. Data set used to estimate the size of bottlenecks during PSbMV seed transmission.

The seedlings obtained from 12 plants (six for inoculum 1 with 38% of variant DPD1-R and six for inoculum 2 with 66% of variant DPD1-R - *i.e.* only from mothers plants having nine of more infected seedlings) were analyzed by ELISA and PSbMV-variant-specific RT-PCR in order to distinguishing four categories of seedlings: (i) healthy, (ii) infected by DPD1, (iii) infected by DPD1-R and (iv) infected by both PSbMV variants. In all, 828 seedlings have been analysed. For each plant, the mean and standard deviation of the relative frequency of DPD1-R variants during the flowering period were estimated using in all 6 leaves, the 3 leaves sampled at 22 dpi and the 3 sampled at 61 dpi. For each plant r ($1 \leq r \leq 12$), these mean and standard deviation estimated were used to calculate the parameters α_r and β_r of the Beta distribution modeling the variability of the proportion of virus variant DPD1-R during the time of seed infection (see Text S1).

Plants	Inoculum	Relative frequency of DPD1-R variant		Total number of seed	Seedlings categories			
		Mean	SD		(i) Healthy	(ii) DPD1 only	(iii) DPD1-R only	(iv) Both variants
1	1	0.32	0.033	23	14	2	4	3
2	1	0.26	0.017	63	39	11	5	8
3	1	0.3	0.015	63	46	7	6	4
4	1	0.34	0.022	70	46	11	5	7
5	1	0.35	0.051	85	48	13	12	10
6	1	0.35	0.044	74	45	15	7	7
7	2	0.65	0.031	70	49	4	15	2
8	2	0.5	0.04	95	43	11	22	18
9	2	0.47	0.073	75	55	3	8	9
10	2	0.52	0.051	53	37	5	3	8
11	2	0.56	0.057	70	53	0	10	6
12	2	0.6	0.061	87	62	7	15	3
