# Supplementary material for: Narrow Bottlenecks Affect Pea Seedborne Mosaic Virus Populations during Vertical Seed Transmission but not during Leaf Colonization
Source: PLoS Pathog. 2014 Jan 9;10(1):e1003833. doi: 10.1371/journal.ppat.1003833 (PMC3887104; doi:10.1371/journal.ppat.1003833)
Supplement: Text S1 — Identifiability of the virus vertical transmission models. (PDF) [file ppat.1003833.s003.pdf]

Text S1. Identifiability of the virus vertical transmission models

Because model identifiability was difficult to establish theoretically, we evaluated the identifiability of parameters by a simulation approach as follows. First, for a given model M_j, $\Lambda = \left(\lambda^1, \lambda^2, \lambda^c\right)$ ($\Lambda = \left(\lambda^1, \lambda^2\right)$ for model M4) was independently drawn from uniform distributions in [0, 25]. Next, datasets similar to our experimental design (*i.e.* R=12 plants and S_r =68 seeds per plant) were simulated given Λ. For each plant *r*=1,...12, we first randomly draw the mean μ_r (respectively the standard deviation σ_r) of the relative frequency of virus variant 1 according to a Beta(5,5) (respectively to a Uniform(0.01,0.1)) distribution. Then, conditionally on μ_r and σ_r, the parameters α_r and β_r of the Beta distribution modeling the variability of the proportion of virus variant 1 during the time of seed infection (*i.e.* from 22 to 61 dpi) were calculated as $\alpha_r = \left(\frac{1-\mu_r}{\sigma_r^2} - \frac{1}{\mu_r}\right)\mu_r^2$ and $\beta_r = \alpha_r\left(\frac{1}{\mu_r} - 1\right)$. Next, we randomly draw a relative proportion Φ_{rs} of variant 1 for each seed *s*=1,…,68 in the beta distribution $\text{Beta}\left(\alpha_r, \beta_r\right)$. Finally, Poisson-distributed random variables $N_{rs}^1 \sim Poisson\left(\lambda^1 \Phi_{rs}\right)$, $N_{rs}^2 \sim Poisson\left(\lambda^2\left(1-\Phi_{rs}\right)\right)$ and $N_{rs}^c \sim Poisson\left(\lambda^c\right)$ were drawn to determine the infection status $X_{rs} = (X_{rs}^{00}, X_{rs}^{01}, X_{rs}^{10}, X_{rs}^{11})$ of seed *s* according to the rules described in Table 2 for the 4 models.

Identifiability depended strongly on the range of parameters $p_{r,\Lambda}^{ij}, i, j \in \{0,1\}$. The farther $p_{r,\Lambda}^{ij}$ from the boundary values 0 and 1 (or the boundary of the simplex domain parameter), the better the model identifiability. Consequently, we restrained our identifiability analysis to the simulated datasets for which the empirical proportions of infected seeds $\overline{X^{ij}} = \sum_{r=1}^{12}\sum_{s=1}^{68} X_{rs}^{ij} \Big/ 12 \times 68$ exceeded 0.05 for the three categories of infected seeds and exceeded 0.2 for uninfected seeds. All these steps were iterated until 100 datasets satisfying the previous constraints were obtained. Then, $\Lambda = \left(\lambda^1, \lambda^2, \lambda^c\right)$ ($\Lambda = \left(\lambda^1, \lambda^2\right)$ for model M4) was estimated using the maximum-likelihood method and compared to the initially drawn “true” values. A high correlation between true and estimated parameters indicates a good practical identifiably. All computations were performed with the R software environment (http://cran.r-project.org/). Parameter inference was performed by minimizing the log of the likelihood

function $\ell_{M_j}(\Lambda) = -\log\left(L_{M_j}(\Lambda)\right)$ (see Materials, methods and models parts) for each model M_j using the “bbmle” package with the “nlminb” optimization routines. 95% confidence intervals for model parameters were estimated using the function “profile” of the “bbmle” package.

Numerical simulations were also designed to check if AIC (Akaike Information Criteria)-model based selection identified efficiently the “true” model that has been used to generate the data. We proceeded as indicated above, except that we fitted to each dataset the true model (as previously) and also the 3 alternative models. The four models were then compared using AIC estimated as $2k - \ln(\ell)$, where k is the number of parameters of the model considered and ℓ is the maximized value of the likelihood function. Model selection efficiency was assessed as the proportion of cases where the true model was selected.
